# Heart rate variability in patients with atrial fibrillation of sinus rhythm or atrial fibrillation: chaos or merit?

**DOI:** 10.1080/07853890.2025.2478474

**Published:** 2025-03-13

**Authors:** Lifan Zhang, Bingxun Li, Lin Wu

**Affiliations:** Department of Cardiology, Peking University First Hospital, Beijing, China

**Keywords:** Heart rate variability, atrial fibrillation, cardiac nervous system, outcome

## Abstract

Atrial fibrillation (AF) is the most common sustained cardiac arrhythmia characterized by consistently irregular atrial and ventricular contractions. Heart rate variability (HRV) refers to the changes in the intervals between consecutive ventricular heartbeats. In sinus rhythm, HRV may be subtle and is quantitatively reflecting the dynamic interplay of the cardiac autonomic nervous system, which plays a crucial role in the onset, development, and maintenance of AF. HRV metrics, consisting of time-domain, frequency-domain, and nonlinear parameters, have been verified to vary significantly before and after AF episodes, and AF treatment-related procedures such as electrical cardioversion, ablation, and surgery of AF. Therefore, HRV may serve as a digital biomarker in predicting AF risk in long-term and acute risk period, identification of patients with AF risk in sinus rhythm and recurrence risk stratification after procedures. HRV in AF rhythm, predominantly influenced by dynamic atrioventricular node conduction under the onslaught of irregular atrial impulses, shows a huge disparity compared to that in sinus rhythm. Despite this, HRV in AF rhythm still provides valuable prognostic information, as reduced HRV may indicate a poor heart function and outcomes in patients with AF. Despite being influenced by lots of variables, HRV can still serve as an independent digital biomarker in the clinical management of AF throughout its entire lifecycle.

## Introduction

Atrial Fibrillation (AF) is the most common sustained cardiac arrhythmia, characterized by consistently irregular atrial and ventricular contractions with increased morbidities and mortality [1]. The prevalence of AF is steadily increasing in recent years, with the lifetime risk reaching up to 1 in 3 as the population in aging and monitor technologies in improving [2,3]. Inadequately treated patients with AF may suffer severe complications such as progressive heart failure, stroke/transient ischemic attack (TIA), and sudden cardiac death, putting a huge burden on social health system [4,5]. Therefore, it is essential to explore and implement additional methods for AF management in clinical practice.

Heart rate variability (HRV), which refers to fluctuations in the intervals between each heartbeat, has become a focal point in recent decades, as it serves as an independent variable reflecting heart-brain interactions [6]. Autonomic imbalance has been identified to be a strong modulator of AF and a potential therapeutic target [7]. Thus, HRV may provide a digital reflection of the autonomic nervous system’s (ANS) impact on AF. However, it must be acknowledged that a significant gap remains before HRV can be fully integrated into the clinical management of AF. This manuscript centers on current clinical evidence and proposes HRV as an independent biomarker for clinical management of AF throughout its entire lifecycle, including predicting AF risk, determining AF history, assessing recurrence risk following catheter ablation, and evaluating long-term prognosis in AF rhythm.

### HRV

Most of the time, it is difficult to perceive the subtle variations between each beat of a seemingly normal rhythmic heart primarily through palpation or auscultation. However, in response to sudden and constant physiological or psychological changes of milieu interne, a healthy heart needs to adjust each beat swiftly. HRV specifically refers to the subtle changes in the time intervals between consecutive heartbeats, called interbeat intervals (IBIs) [8]. Given the situation of atrioventricular asynchrony like AF or atrioventricular block which is different from that in sinus rhythm, HRV mentioned in this paper solely refers to ventricular rate variability. By analyzing a continuous electrocardiogram (ECG) recording, ranging from 10 s to over 24 h, we can yield a series of HRV time-domain, frequency-domain, and nonlinear parameters [9].

HRV time-domain parameters are derived from quantitative calculations of IBI variability, in which the most commonly used indices include the standard deviation of normal-to-normal (NN) intervals (SDNN), the root mean square of successive NN interval differences (RMSSD) and percentage of successive NN intervals that differ by more than 50 ms (pNN50). SDNN necessitates a minimum 5-minute ECG recording and is impacted by both sympathetic and parasympathetic nervous system [10]. SDNN derived from ECG recordings exceeding 24 h at sinus rhythm has been proven to be correlated with morbidity and mortality. A cohort study involving survivors of acute myocardial infarction (AMI) showed that patients with SDNN less than 50 ms had an all-cause mortality rate 5.3 times greater than those with SDNN greater than 100 ms within a median follow-up period of 31 months [11]. RMSSD and pNN50 accurately reflect parasympathetic nervous system activity, requiring at least a 5-minute and a 2-minute epoch, respectively, with the former being less affected by respiration [12,13].

By using fast Fourier transform, the oscillations of heart rate can be categorized into different frequency-domain metrics, including ultra-low frequency (ULF), very-low frequency (VLF), low frequency (LF), and high frequency (HF) bands, analogous to electroencephalograms [9]. HF band, known as the respiratory frequency domain due to its close association with respiratory sinus arrhythmia (RSA), shows a close correlation with RMSSD and pNN50, all of which reflect the parasympathetic nervous system activity [14]. Frequency domain parameters can be normalized or expressed as ratios because of the huge difference among healthy individuals even matched with age. Noteworthily, the previous belief that the ratio of LF to HF reflected sympathetic/parasympathetic balance, where an increase in the ratio indicated heightened sympathetic activity accompanied by parasympathetic withdrawal is being challenged due to limited correlation between sympathetic tension and the LF band, as well as the non-linear, non-reciprocal relationship between the sympathetic and parasympathetic nervous system [15].

The Poincaré plot, applied by fitting adjacent RR intervals alternately as the x and the y, coordinates to form an ellipse and derives lots of nonlinear parameters, including SD1, SD2 and SD2/SD1. SD1 reflects short-term changes in heart rate and therefore represents vagal tone because the parasympathetic nerve dominates for rapid changes in heart rate. SD2 and SD2/SD1 are strongly correlated with LF/HF, representing the balance between long- and short- term HRV [16–18].

HRV triangular index (HRVI) is calculated as the total number of NN intervals divided by the number of NN intervals with the highest proportion. HRVI is less likely affected by artifacts or noises [19]. Reduced HRVI reflects autonomic imbalance, while it does not provide information about specific changes in sympathetic or parasympathetic tone [20]. In the field of feature extraction, approximate entropy, sample entropy, and multiscale entropy are widely used. These metrics are initially used to characterize the complexity and unpredictability of time series where larger entropy values indicate greater complexity.

### Anatomy of autonomic nervous system

The heart is under the joint control of the intrinsic and extrinsic branches of the ANS. Central ANS signals originate from several areas including cerebral cortex, cingulate cortex, amygdala, thalamus [21]. Sympathetic fibers descend to the intermediolateral column of the spinal cord, in which preganglionic neurons project to paravertebral ganglia like cervical, thoracic, stellate and mediastinal ganglia. There postganglionic noradrenergic neurons extend long axons to epicardial ganglia or directly to cardiac cells [22]. In the contrary, preganglionic fibers of the parasympathetic nervous system derived from brainstem, mainly nucleus accumbens and dorsal motor nucleus of the medulla oblongata, travelling along vagal nerve, ultimately target the short-axon postganglionic neurons in the cardiac ganglia [23].

The intracardiac nervous system is a complex neural network composed of ganglionated plexuses (GPs) embedded within the epicardial fat pads and the wall. It encompasses an extensive array of sympathetic and parasympathetic nerve endings, playing a role in integrating signals of the ANS [24]. Approximately 75% of the epicardial ganglia are located at the back of the heart, with the highest density within the fat pad at the junction of the left atrium and pulmonary veins [24].

### ANS and AF

Autonomic imbalance, either the activation of the sympathetic or vagal components, plays a crucial role in the onset, development, and maintenance of AF [7].

When sympathetic activation occurs for various reasons, postganglionic sympathetic neurons secret excessive norepinephrine to act on adrenergic receptors on atrial myocytes. Through the protein kinase A-mediated adenylate cyclase/cAMP signaling pathway, the sarcolemmal L-type calcium current is phosphorylated, leading to enhanced transmembrane Ca^2+^ inflow during the plateau phase of the action potential. Increased calcium in dyadic cleft between the t-tubules and sarcoplasmic reticulum (SR) induced opening of ryanodine receptors 2 (RyR2) on the SR, resulting in an elevated intracellular calcium load, i.e. Ca^2 +^ induced Ca^2 +^ release. Simultaneously, calcium overload activates Ca/Calmodulin-dependent kinase II (CaMKII), initiating a series of cascading reactions. PKA and CaMKII phosphorylate RyR2, augmenting its calcium release capacity and thereby intensifying excitation-contraction coupling [25,26]. Despite SR Ca^2 +^ ATPase’s efforts to mediate calcium reuptake into SR, calcium overload ultimately occurs within both cytosol and SR [27,28]. Calcium leakage mediated by RyR2 *via* the store-overload-induced calcium release mechanism also plays a significant role in this process. To extrude excessive calcium, sodium-calcium exchangers on the sarcoplasmic membrane begin to function in a forward mode, generating a net inward current to depolarize membrane potential, i.e. delayed afterdepolarization [7,27,28]. In addition to atrial electrical remodeling, long-term sympathetic activation, while increasing myocardial contractility, may also lead to calcium-induced hypertrophy and fibrosis [29].

In structurally normal hearts, parasympathetic components dominate. Acetylcholine (ACh) released from the vagal nerve can activate ACh-sensitive potassium channels (I_K-ACh_) on atrial myocytes and promote repolarization, thereby heterogeneously shortening the action potential duration of atrial myocytes, eventually facilitating reentry and atrial arrhythmias [30]. Besides, the vagal nerve can also act as a trigger of AF. Due to shorter atrial effective refractory period, atrial tachycardia is permitted to occur. When tachycardia is terminated, intracellular calcium overload can induce delayed and early afterdepolarization [31].

ANS promotes trigger activities and reentry, leading to atrial structural remodeling, and is of significant importance in the pathogenesis and progression of AF. HRV, as an indicator of autonomic nervous function, may serve as a significant biomarker for AF.

### HRV before AF onset

As mentioned above, ANS tone is one of the most crucial regulatory factors in atrial electrical activities, referring to changes of ion channel function and triggering activities [32]. Autonomic imbalance is frequently seen prior to AF onset, reflected in notable alterations in HRV parameters before AF episodes [33,34]. An abrupt shift towards vagal predominance in an augmented adrenergic tone milieu may precipitate AF occurrence, manifesting as a significant increase in HF band and a simultaneous gradual decrease in LF band, whether it is ‘lone’ AF or accompanied by overt structural heart disease [33]. Adrenergic activation and vagal withdrawal appear to contribute to the onset of postoperative or exercise-induced AF. Before these types of AF, there is a notable decrease in RR interval and significant escalating time- and frequency-domain indices [34]. In various scenarios, the predominant influence of any branches of ANS system can precipitate the occurrence of AF. Hence, a consistent and aligned shift in various HRV parameters may serve as a digital biomarker of an elevated risk of AF.

### HRV in patients with AF rhythm

HRV in AF rhythm differs significantly from that in sinus rhythm, exhibiting notably greater irregularity and unpredictability in which the irregular changes in beat-beat intervals can be easily observed through auscultation or an ECG. In contrast to sinus rhythm, where HRV is primarily modulated by the ANS, irregular ventricular contractions in AF rhythm are predominantly influenced by atrioventricular node function. Frequent and irregular atrial impulses alter the refractory period of the atrioventricular node through concealed conduction, thereby impacting its conduction function and resulting in extremely irregular ventricular contractions [35]. With fluctuations in atrial rates, AV synchrogram analysis reveals various n:m coupling patterns of atrioventricular activity (ranging from 2:1 to 7:1) [36]. As the atrial rate rises, the atrioventricular conduction function worsens. It is further observed that rapid and irregular atrial impulses impacting the atrioventricular node might cause more pronounced concealed conduction, consequently leading to heightened irregularity in the ventricular rates [36–38].

Vagal nerve can also regulate atrioventricular node conduction. Ablating GPs to denervate vagal nerve can improve functional atrioventricular conduction block [39,40]. Stimulating the vagal nerve at the atrioventricular node has been proven to slow ventricular rates in AF rhythm and reduce inappropriate ICD discharges due to rapid atrial arrhythmias. Yet, the relationship between ANS and HRV in AF rhythm remains unclear. Varying vagal nerve stimulation intensities and sites seem to elicit diverse changes in HRV. An experiment shows that lower level vagal nerve stimulation at the fat pad of junction of the inferior vena cava and the left atrium increases HRV mildly, while stronger stimulation at the same site decreases it close to baseline levels [41]. When stimulating vagal nerve at the posterior aspect of right atrium, HRV is completely eradicated [42].

## HRV as a predictor of AF risk

### HRV and the risk of long-term incident of AF

Decreased HRV in sinus rhythm, reflecting autonomic imbalance characterized by hyperactive sympathetic system and a hypoactive parasympathetic tone, has been identified as a risk factor for a range of cardiovascular diseases (CVD) [43]. However, HRV in sinus rhythm among general population shows an ambiguous association with the onset of AF. Both excessive and decreased HRV parameters might be associated with an increased risk of AF.

In the Atherosclerosis Risk in Communities (ARIC) cohort study, the relationship between HRV and the risk of incident AF was explored in a group of 11715 middle-aged adults with an average follow-up of 19.4 years. Cardiac autonomic dysfunction denoted by low resting short-term HRV indices (including SDNN and HF) obtained from 2-minute resting heart rate data was associated with higher AF incidence [44]. This conclusion hasn’t been consistently validated in subsequent studies. High HRV parameters such as SDNN and RMSDD, indicating elevated vagal tone, appear to be similarly associated with an elevated incidence of AF [45,46]. In the population-based Rotterdam study, an analysis of baseline 10-second electrocardiogram data from 12,334 participants free of AF revealed an association between elevated SDNN and RMSDD parameters and the risk of incident AF, which is more prominent among women [46]. Further analysis within the Multi-Ethnic Study of Atherosclerosis (MESA) cohort, involving 6,261 participants free of AF and diagnosed CVD, discovered a correlation between both excessively high and low HRV parameters and higher risk of incident AF [47].

Study conclusions may vary due to differences in study populations and HRV measurement methods. The retrospective study involved hypertensive patients with a higher prevalence of diabetes, coronary artery disease, and AF history than the ARIC and MESA cohort [45]. HRV was derived from various ECG data, including 10-second and 2-minute ECGs or 24-hour Holter monitoring. While some HRV metrics are comparable in different time scales, it may still reach or obscure certain outcomes [48]. Besides, HRV reflects the degree of ANS fluctuations and deviations from autonomic balance and the extent of deviation may matter more than its direction over longer periods. Furthermore, over extended periods, a single baseline HRV may not accurately reflect long-term autonomic imbalance. Focusing on short-term HRV metrics can offer better predictive value, as demonstrated in the retrospective study, which, despite a small sample size and short follow-up, revealed a strong association between HRV and AF [45].

HRV is influenced by multiple confounding variables [6]. It is well-established that HRV can assist in the early diagnosis and severity assessment of infection. Prior to the onset of sepsis, several HRV parameters have been observed to decrease significantly, and persistently low LF has been associated with disease deterioration and mortality [49]. In recent years, increased HRV metrics including RMSSD, pNN50 and HF, a tendency toward parasympathetic overtone have been welled documented in post-COVID patients [50]. Moreover, a recent meta-analysis revealed a significant increased risk of incident AF after COVID-19 infection, which may be partly attributed to cardiac autonomic dysfunction [51].

### HRV and the risk of AF in the acute phase of CVD

AF frequently occurs in patients in acute stages of various cardiovascular conditions, such as AMI and ischemic stroke. Concomitant AF often suggests a poorer prognosis in these patients than the normal rhythm [52,53]. HRV performs better in predicting the risk of AF in sinus rhythm, especially during the short-term high-risk phase. As AF approaches, significant changes in ANS tone lead to notable alterations in HRV parameters [54]. In a retrospective analysis of baseline 24-hour Holter recordings in hospitalized patients suffering AMI, it was observed that patients who developed AF post-heart attack showed a decreased LF/HF ratio, increased RMSSD and pNN50 in baseline sinus rhythm compared to those free of AF [55].

Extended electrocardiographic Poincare analysis (EPA), also called stroke risk analysis (SRA) algorithm, holds value in predicting the risk of AF in different conditions [56]. The algorithm derives predictive insights for AF risk stratification by utilizing dynamic parameters from RR intervals, involving visual analysis of the Poincaré plots and a decision matrix based on multiple HRV linear and nonlinear parameters like principle component analysis of R-R interval data, calculated standard deviations of R-R interval data, number of premature atrial complexes without sinus node reset, etc [56]. Using patient’s 48-hour baseline continuous cardiac monitoring data following admission to the stroke unit, SRA algorithm effectively predicts the risk of paroxysmal AF in patients with acute ischemic stroke or TIA (high-risk 38.5% versus low-risk 0.9%) [53]. SRA algorithm can also serve a biodigital biomarker to predict the risk of AF post coronary artery bypass grafting surgery among individuals with coronary artery disease. When analyzing ECG data from last three hours before AF onset, SRA yields a higher accuracy, suggesting changes of HRV parameters under automatic imbalance precede AF episodes [54].

## HRV in AF identification

### HRV in AF rhythm and AF diagnosis

Early identification and appropriate management of AF may prevent severe deteriorating complications associated with AF like stroke and heart failure. The 2020 European Society of Cardiology (ESC) Guidelines for the Diagnosis and Management of Atrial Fibrillation recommend that diagnosing AF requires either a standard 12-lead ECG recording or a single-lead ECG tracing of ≥30 s, displaying an irregular heart rhythm with no distinct repeating P waves and irregular RR intervals [57]. Certain AF patients exhibit episodes devoid of overt symptoms, like palpitations or dyspnea, categorized as subclinical AF. Nevertheless, individuals with subclinical AF still face an increased risk of stroke and potential disease progression leading to heart failure [58]. Therefore, early screening for high-risk AF patients is imperative.

Technological advancements in wearable monitoring devices utilizing artificial intelligence or machine learning (MR) have emerged as pivotal breakthroughs in the early identification of AF. Photoplethysmography (PPG) is an optical method that uses a mobile phone-based optical sensor to measure subtle changes in the pulse wave during cardiac contraction/dilation without requiring additional external devices. Several large-scale clinical studies have demonstrated the efficacy of PPG-based AF screening strategies. However, those PPG-based screening approaches exhibit a positive predictive value ranging from 50 to 80%, which could lead to false-positive identifications [59,60]. Furthermore, PPG signals during physical activity may encounter heightened background noise and artifacts, potentially making the diagnosis of AF more challenging and less reliable [61].

The substantial disparity of HRV between AF and sinus rhythm underscores the important role of HRV in AF identification. Among the various proposed ECG-based algorithms for AF identification, ventricular features, i.e. ventricular rate variability, are the most commonly applied. As machine learning and artificial intelligence progress rapidly, these algorithms, derived from HRV linear, nonlinear parameters to neural networks trained directly on ECG data, are becoming increasingly effective, yet more complex and less well interpretable. In terms of linear measures, pRRx (the percentage of consecutive RR intervals that differ by more than x (ms) and pRRx% (the percentage of consecutive RR intervals that differ by more than x %) appear to be the most effective to distinguish AF from sinus rhythm [62,63]. Leveraging extensive machine learning and maximum-relevance minimum-redundancy algorithms, the optimal ML classifier including pRR50, SD2/SD1), and RR interval variability coefficient achieves an accuracy of 97.2% [63]. An algorithm solely derived from pRR3.25% also exhibits excellent diagnostic properties for detecting AF [62]. HRV nonlinear metrics such as Poincaré plot, entropy measures like sample entropy, coefficient of the sample entropy, Shannon entropy, and turning point ratio are widely utilized as they reflect the inherent unpredictability of RR intervals [64]. When combined with effective machine learning methods such as k-nearest neighbor classification, support vector machines, most models based on these nonlinear metrics demonstrate comparable detection efficacy [64,65].

## HRV for identifying paroxysmal AF patients in sinus rhythm

AF episodes can be paroxysmal and sometimes asymptomatic. When patients present with sinus rhythm during medical evaluation, it may delay diagnosis and potentially lead to serious complications. Continuous electrocardiographic monitoring for all individuals at high risk of stroke would greatly waste medical resources. Therefore, it is crucial to implement a non-invasive, low-cost method to identify patients with a history of AF.

Some studies have shown that electrocardiographic features of sinus rhythm, such as PR interval, P-wave dispersion, and P-wave signal averaging, may be indicative, while these individual features still lack sufficient diagnostic value to be further utilized in clinical practice [66]. An artificial intelligence-enabled ECG algorithm could utilize ECG in sinus rhythm to screen patients with a history of AF or at risk of developing AF, with higher sensitivity compared to usual care [67,68].

HRV in sinus rhythm can also assist in identifying patients with a history of AF [56]. During an average of 19.5 h of cardiac monitoring, conventional long-term electrocardiographic monitoring detected AF episodes in only 13.8% (4/29) of patients with confirmed AF history. In contrast, EPA analysis based on a variety of HRV linear and nonlinear parameters exhibited a higher sensitivity of AF history. Specifically, 89.7% of patients with AF history were classified as either risk level 1 (indicating deviation from sinus rhythm and potential risk of AF) or risk level 2 (suggestive of AF). Meanwhile, 19.0% of healthy controls were misclassified as risk level 1 by EPA analysis. Further research is needed to confirm the role of EPA in using HRV at sinus rhythm to identify AF. In real-world scenarios, patients often have more comorbidities and inconsistent HRV, potentially leading to increased misclassifications with EPA analysis.

## HRV and the prognosis in patients with AF

### Post-procedure HRV parameters: correlating with the risk of AF recurrence

Due to the highest density of GPs within the fat pad at the junction of the left atrium and pulmonary vein ostia, procedures near the pulmonary vein ostia, whether utilizing radiofrequency ablation or cryoablation, are always accompanied by significant changes in ANS tone. In the preliminary stages, when performing focal ablation within the pulmonary vein in paroxysmal AF patients, researchers found some might experience severe hypotension or bradycardia, which was considered as vagal reflex [69]. Postoperatively, patients receiving pulmonary vein ablation show a notable increase in average heart rate, along by a significant decrease in HRV parameters reflecting vagal tone, compared to preoperative levels [70]. Later, Pappone et al. identified that abolition of all evoked vagal reflexes around all pulmonary vein ostia to achieve complete vagal denervation could significantly reduce recurrence risk of AF [71], suggesting that achieving complete vagal denervation might be a vital mechanism in treating AF through circumferential pulmonary vein ablation, rather than merely a side effect. Similarly, post-cryoballoon ablation, the elevated vagal nerve activity postoperatively, as reflected by HRV parameters, strongly suggests pulmonary vein reconnection [72].

Based on this, several studies have consistently concluded that HRV parameters in sinus rhythm post-ablation may serve as a robust predictor for the recurrence risk of AF. In a study of 102 patients with paroxysmal AF who underwent radiofrequency ablation, those in the late recurrence group during a 24-month follow-up had higher HRV parameters (RMSSD, pNN50, and HF) on the first day postoperatively compared to the non-recurrence group. This suggests that late recurrence after radiofrequency ablation can be partly attributed to an elevated level of vagal nerve activity postoperatively [73]. Marinković and colleagues quantitatively illustrated the trend in changes of HRV parameters during follow-up in patients with AF undergoing radiofrequency ablation. Post-ablation, there is a significant decrease in HRV parameters related to vagal activity, and these parameters gradually returned to preoperative levels during the follow-up period. Notably, 3‑month post‑CPVI SDNN reduction still retained predictive value for late recurrence of AF [74]. Incorporating procedures on the pulmonary veins and left atrium, successful surgical ablation of AF, like the modified Maze III procedure or CPVI, also exhibit a similar pattern in the changes of HRV parameters [75].

These findings collectively suggest a crucial role of heightened vagal nerve activity in the onset and maintenance of AF, especially when involving pulmonary veins. Moreover, they validate the utility of HRV-related parameters to predict post-ablation recurrence of AF.

As catheter ablation becoming a high-level recommended option for patients with AF and heart failure to reduce heart failure hospitalization, and potentially reverse left ventricular dysfunction caused by tachycardia-induced cardiomyopathy [1,76]. It is crucial to select patients who will benefit most from AF catheter ablation and repeat catheter ablation [77]. CPVI terminates AF in part by achieving complete vagal denervation. Parasympathetic overtone indicated by pre-ablation HRV metrics may suggest that these patients are more appropriate candidates for first-time or repeat AF catheter ablation, requiring further research to evaluate. In addition, inflammation, related to HRV, has been shown to predict both the onset and recurrence of AF [78,79].

However, the relationship between HRV parameters after electrical cardioversion and the risk of AF recurrence remains unclear, potentially affected by a variety of antiarrhythmic drugs being used. Lombardi et al. observed that short-term HRV parameters measured 4–5 h post cardioversion can predict the risk of AF recurrence in persistent AF patients receiving amiodarone. A higher LF/HF ratio and lower HF suggest an increased AF reoccurrence risk during the first two weeks after cardioversion [80]. In another study involving patients with persistent AF who were partially treated with beta-blockers and class Ic antiarrhythmic drugs, increased HF power spectral component, referred to enhanced vagal outflow, was the strongest predictor of early recurrence after cardioversion [81]. Although AF recurrence after cardioversion is partly linked to ANS dysfunction, the precise relationship with HRV parameters remains unclear, which limits its clinical application.

### HRV and the stroke risk

While low nocturnal or 24-hour HRV parameters in sinus rhythm may indicate a higher stroke risk in individuals without CVD history [82,83], their relationship with stroke risk in AF remains unclear.

Traditional HRV linear parameters, along with non-linear ones mentioned above, appear to fall short to predict ischemic stroke events in AF patients. A cross-sectional study of 1,358 AF patients without a history of stroke/TIA found that impaired HRV in AF rhythm was associated with a higher incidence of brain infarcts detected by MRI. In sinus rhythm subgroup, impaired HRV also indicated a larger infarct volume, predominantly in the right cerebral hemisphere. However, it does not imply that impaired HRV metrics lead to an increased risk of stroke. The relationship between HRV in sinus rhythm and the extent and location of infarction suggests that the ANS is affected by the infarction itself [84]. In another cohort study, baseline 5-minute ECG data from AF patients revealed that a reduced HRVI below 14.29 predicted overall or cardiovascular mortality over a 2.6-year follow-up, but not the risk of ischemic stroke or TIA [85].

When focusing on more complex nonlinear metrics, multiscale entropy, an expansion from sample entropy in different time scales, has been found to be linked to the incident stroke risk in patients with persistent AF. Rises in the average value of multiscale entropy within the VLF subrange (90–300 s, MeanEnVLF2) appears to be paralleled increase in risk of acute stroke during a median follow-up period of 48 months. In contrast, no effect was observed among patients receiving antithrombotic drugs, including warfarin or antiplatelet drugs [86]. The heightened visit-to-visit heart rate variability (VVV-HR), quantified as the standard deviation of heart rate data across all visits, emerges as an independent prognostic factor for adverse outcomes in AF patients, including overall mortality, stroke/TIA, and heart failure risks [87]. Differing from HRV parameters calculated from minutes or hours of RR intervals, VVV-HR reflects poor treatment adherence, inadequate symptom/substrate management, or heightened sympathetic activity [87].

### HRV in AF rhythm and prognosis

AF-induced cardiomyopathy is not solely attributed to tachycardia commonly observed in most cases. The irregularity of AF rhythm detrimentally affects ventricular substrate. At the cellular level, *in vitro* experiments demonstrate that irregular ventricular pacing leads to diminished calcium transient amplitudes and sarcoplasmic calcium load, resulting in a decrease of ventricular contractile capacity [88,89]. Besides, irregular rhythm promotes increased secretion of pro-fibrotic cytokines like CTGF, TGF-β, and oxidative stress, contributing to adverse ventricular remodeling [88,89]. At the nervous activity level, by irregular pacing in the right atrium mimicking acute ventricular response in AF rhythm, sympathetic activity remarkably increased compared with a regular ventricular pacing [90]. Meanwhile, irregular ventricular contractions consistently signify poorer hemodynamic performance. AF patients exhibit significant improvement in hemodynamics after electrical cardioversion, despite adequate control of ventricular rates before [91].

However, the relationship between HRV parameters in AF and the prognosis appears to be in stark contrast to that observed *in vitro* experiments. A small cohort study conducted in the 1990s found that SDANN less than 100 ms in chronic AF with severe heart failure suggested a poorer prognosis [92]. Yamada et al. later found among persistent AF patients, decrease HRV non-linear metric ApEnb-b was an independent predictor (*p* = 0.04) of 5-year cardiovascular mortality risk, but not traditional time-domain parameters (e.g. SDNN, SDANN) [93]. Recently, the multicenter prospective Swiss-AF cohort study revealed a correlation between reduced HRVI and heightened risks of cardiovascular and overall mortality in AF patients, irrespective of their baseline rhythm being sinus or AF [85]. While most studies suggest an adverse outcome with diminished HRV, when focusing on HR range (defined as the difference between HR maximum and HR minimum derived from a 24-hour ECG), it was found that a larger HR range was associated with heart failure events among patients with permanent AF. While reduced HRV in AF rhythm is generally linked to poor outcomes, patients with permanent AF and a larger HR range (defined as the difference between HR maximum and HR minimum derived from a 24-hour ECG) may experience more heart failure events future [94].

Although irregular rhythm impairs contractile function, disrupts autonomic balance, and deteriorates hemodynamics, HRV itself might not have a direct predictive value on prognosis. Conversely, the upstream regulatory factors of HRV could play a pivotal role in predicting the prognosis. Multiple factors collaboratively regulate HRV in AF rhythm, including autonomic tone, concealed conduction of AV node, and atrial and ventricular structural remodeling. Non-invasive assessment of post-AF atrial rate reveals that a lower atrial dominant frequency may indicate more extensive atrial fibrosis [95]. Atrial and ventricular remodeling leads to decreased HRV and, simultaneously, poor prognosis.

Further research is warranted to explore mechanisms underlying the changes of HRV in patients with AF, especially during AF, and how HRV can be further applied in clinical practice.

## Limitations

Although HRV is a valuable biomarker in the clinical management of AF throughout its entire lifecycle, and its acquisition method is non-invasive and relatively simple, it must be addressed that HRV is influenced by multiple confounding variables, including demographic variables, infection, concurrent medications and comorbidities, and even mental status, as well as variations in HRV measurement standards [6,49]. Besides, although relevant studies have reached relatively certain conclusions—such as lower levels of postoperative vagal-related parameters indicating a reduced risk of recurrence after catheter ablation—the parameters themselves differ. The absence of a unified cut-off value, or context-specific thresholds, remains a key factor limiting its broader clinical use. Despite being influenced by lots of variables, HRV has been shown to strongly correlate with AF, further suggesting that this correlation is not merely a statistical artifact under specific conditions, but rather reflects an underlying physiological mechanism, specifically changes in ANS tone.

HRV might be better applied in the clinical practice of AF through the following ways: (1) Control modifiable confounding factors and standardize measurement protocols. (2) Utilize parameters less influenced by confounding factors, such as HRVI and standardized frequency-domain HRV parameters; establish distinct cut-off values for various comorbidities. (3) Focus on short-term changes in HRV parameters to minimize the influence of confounding factors, with autonomic nervous system tone as the primary determinant. Further clinical research is required to explore and validate these approaches.

## Conclusion

**Figure 1.**
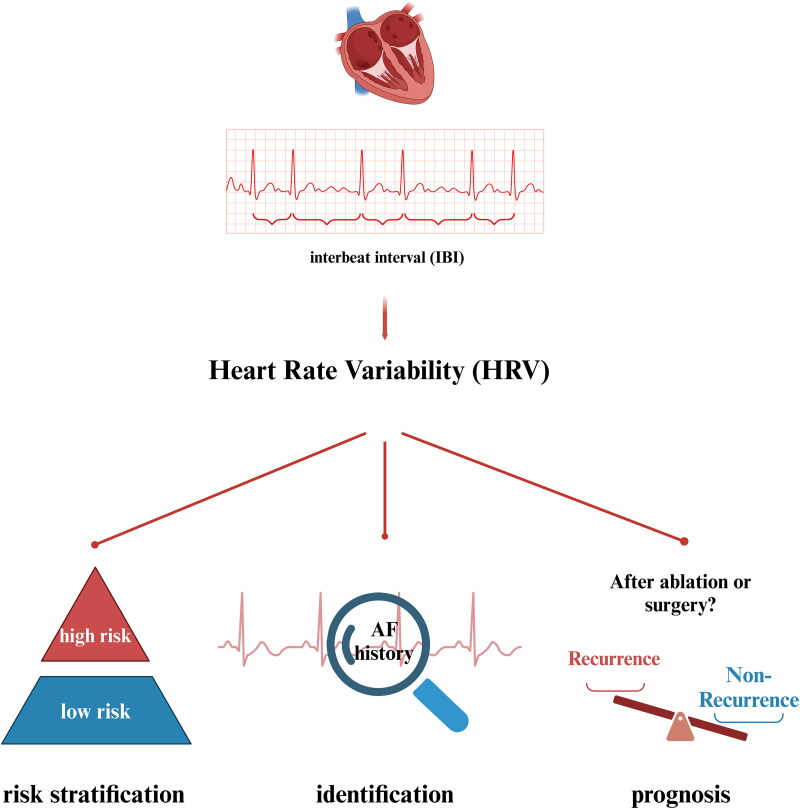
Heart rate variability and atrial fibrillation (created in BioRender. Lifan, Z. (2025) https://BioRender.com/b68f835).

HRV analysis in patients with AF, both in sinus rhythm and during AF rhythm, provides values in risk stratification, identification and prognosis in patients with tendency to be AF or with AF (Figure 1).

Due to the notable heterogeneity in real-world AF populations, characterized by variations in AF burden, comorbidities, approaches to rhythm or rate control, coupled with substantial differences in the measurement of HRV parameters across studies, further research is needed to improve its application in the management of AF throughout its entire lifecycle.

## Data Availability

No data were generated in this work.
